# Can financial stress be anticipated and explained? Uncovering the hidden pattern using EEMD-LSTM, EEMD-prophet, and XAI methodologies

**DOI:** 10.1007/s40747-022-00947-8

**Published:** 2022-12-26

**Authors:** Indranil Ghosh, Pamucar Dragan

**Affiliations:** 1 IT and Analytics Area, Institute of Management Technology Hyderabad, Shamshabad, Hyderabad, Telangana 501218 India; 2 Department of Operations Research and Statistics, Faculty of Organizational Sciences, University of Belgrade, 11000 Belgrade, Serbia

**Keywords:** Financial stress, Ensemble empirical mode decomposition, Long short-term memory network, Facebook’s prophet algorithm, Explainable artificial intelligence, Technical indicators

## Abstract

Global financial stress is a critical variable that reflects the ongoing state of several key macroeconomic indicators and financial markets. Predictive analytics of financial stress, nevertheless, has seen very little focus in literature as of now. Futuristic movements of stress in markets can be anticipated if the same can be predicted with a satisfactory level of precision. The current research resorts to two granular hybrid predictive frameworks to discover the inherent pattern of financial stress across several critical variables and geography. The predictive structure utilizes the Ensemble Empirical Mode Decomposition (EEMD) for granular time series decomposition. The Long Short-Term Memory Network (LSTM) and Facebook’s Prophet algorithms are invoked on top of the decomposed components to scrupulously investigate the predictability of final stress variables regulated by the Office of Financial Research (OFR). A rigorous feature screening using the Boruta methodology has been utilized too. The findings of predictive exercises reveal that financial stress across assets and continents can be predicted accurately in short and long-run horizons even at the time of steep financial distress during the COVID-19 pandemic. The frameworks appear to be statistically significant at the expense of model interpretation. To resolve the issue, dedicated Explainable Artificial Intelligence (XAI) methods have been used to interpret the same. The immediate past information of financial stress indicators largely explains patterns in the long run, while short-run fluctuations can be tracked by closely monitoring several technical indicators.

## Introduction

Modeling financial stress worldwide through the lens of predictive modeling is of paramount significance owing to consequent implications on routine economic activities [8, 37]. Prediction of financial stress beforehand can assist in anticipating the sudden rise or fall of several critical financial and commodity market variables, including stock indices, crude oil price, etc. It is an extremely arduous task as the evolving time series data reflecting financial stress is bound to exhibit a high degree of a nonlinear and nonparametric pattern. It reflects the ongoing state of the global economy and financial markets subject to uncertainty and volatility of external events. Financial stress can also account for the extent of market fear and sentiment of investors effectively. On the other hand, there exists a high chance of uncertainty and fear arising out of the COVID-19 pandemic to further induce volatility and thereby stress the financial markets in an unprecedented manner. Basically, monitoring the same is primarily meant to inspect whether financial markets behave normally or are being affected by disruptions. Nevertheless, quantifying financial stress is itself challenging. Throughout the literature, attempts have been made to measure the stress penetrating financial markets across geography and assets [10, 25]. Office of Financial Research (OFR) has introduced a daily indicator of financial stress (FSI) embodying different markets [36]. A positive FSI value indicates the prevailing stress level on that day is less than average, whilst a negative figure suggests a lower stress level than average. Barring the standard index, OFR has also monitored the quantum of stress spanning across five categories of indicators, namely, credit (Credit), equity valuation (EV), funding (Funding), safe assets (SA), and volatility (Volatility) and different regions, United States (US), other advanced economies (OAE), and emerging markets (EM). The present work explores the predictability of said assets using LSTM and Facebook's Prophet by performing forecasting exercises. Suppose the future figures can be anticipated well in advance with a high degree of accuracy utilizing the research framework. In that case, an inference can be drawn that the stress in the financial market can be anticipated beforehand and leveraged accordingly.

Research dedicated to modeling the impact of financial stress on heterogeneous assets has received a strong surge of late, as reported in the pertinent literature. Bouri et al. [3] developed a copula-based approach to uncover dependence and causality structure in the quantile between the global financial stress index and bitcoin return. It was revealed that strong evidence of right-tail dependence between the global financial stress index and Bitcoin returns was imminent, while financial stress possessed a significant causal influence on Bitcoin returns. A study by Chen et al. [6] revealed that during a time of high financial stress or when foreign currency observes appreciation, exporting firms would increase export prices and upgrade quality. Research by Rho and Saenz [40] demonstrated that financial stress substantially amplified the effect of debt-to-GDP ratio, stock of international reserves, and GDP per capita on the probability of occurrence of sovereign debt. Qin [38] deployed structural vector autoregression (VAR) to evaluate the impact of structural oil shocks on stress in financial markets and the interaction of different markets. The strong influence of structural oil shocks on both of them was discovered. Liu et al. [31] utilized Markov regime-switching model to comprehend the impact of oil price shock manifested in terms of supply, demand, and risk on the financial stress index of China. Asymmetric and nonlinear interplay structure was extracted. Ozcelebi [34] explored the interaction of the financial stress index of developed countries on the exchange market pressure index of Brazil, China, Mexico, Russia, and South Korea using nonlinear VAR. Findings suggested the presence of a positive association. However, the majority of the existing work has been confined to either evaluating the impact of stress in financial markets on relevant assets or how other potential shocks arising out of different assets affect the stress level. There exists an evident dearth of research on whether financial stress can be predicted or not. It is extremely critical to investigate the temporal dynamics of the financial stress index to check predictability for practical policy implications and regulation to mitigate the adversarial traits. On top of that, an apparent paucity of research dedicated to the predictive modeling of financial stress makes the selection of methodological frameworks extremely difficult. It is also equally important to check the predictability of financial stress during the timeline affected by the COVID-19 pandemic as the same would test the efficacy of utilized research frameworks in modeling high volatile time series. Successful development for robust predictive architecture would be a significant contribution from the methodological point of view as well. Therefore, the development of predictive frameworks capable of estimating short-term and long-term figures of financial stress would be of paramount practical relevance as a close nexus of financial stress with critical macroeconomic and financial indicators has been reported in the literature.

The major contribution of the present work lies in the attempt to rigorously delve into the predictability of financial stress globally through the deployment of advanced predictive modeling architectures. The entire research framework to accomplish the endeavors has been carefully designed and can be considered to be a contribution to pertinent literature from a methodological perspective too. In this work, we have resorted to several financial stress indices monitored by the office of financial research (OFR) for predictive modeling. The said indices have been acknowledged to reflect the quantum of financial stress across different assets and locations in literature. The present work resorts to artificial intelligence (AI) based modeling frameworks to accomplish the research endeavor. The selection of AI-driven modeling frameworks is rationalized for the capability of modeling highly volatile and nonlinear patterns. As hardly any previous research has been done for predictive analytics of financial stress, it is difficult to figure out key determinants of the same. In this work, we have chosen 24 technical indicators as independent features to estimate future movements of financial stress manifested by nine indicators. Technical indicators are basically simple mathematical functions applied to lagged figures of the target construct [7, 15]. They have been successfully used in stock market predictive modeling. The chosen technical indicators have been subjected to the Boruta feature selection algorithm for justifying the deployment of the same. Subsequently, Ensemble Empirical Mode Decomposition (EEMD), a variant of the orthodox Empirical Mode Decomposition (EMD) technique, has been used for disentangling the underlying financial stress series into granular components to capture nonlinearity and volatile counterparts with a high degree of efficacy. Technical indicators utilized as explanatory features have been decomposed too. LSTM model has been used on decomposed components obtained from EEMD to achieve component-wise forecasts. Final forecasts of the combined framework of EEMD and LSTM are obtained by aggregating the component-wise forecasts for respective time series. On the other hand, Facebook's Prophet algorithm has been applied to granular decomposed subseries as well to fetch component-wise forecasts. Likewise, the combined EEMD-LSTM approach final forecasts of the combined methodology of Prophet and EEMD have been obtained by taking arithmetic sum on granular forecasts. The proposed frameworks are fed to a battery performance evaluation to rationalize the quality of forecasts. The underlying work follows the time series forecasting evaluation scheme of Bou-Hamad and Jamali (2020), wherein one step ahead static forecasting capability and multistep ahead dynamic forecasting capabilities are assed. The said setup is useful for evaluating the degree of predictability of short and long-run scales simultaneously. To rationalize the effectiveness of both EEMD-LSTM, and EEMD-Prophet forecasting structures, stringent validation by subjecting the models to extremely distress periods and on surrogate series and comparative statistical assessment against a set of benchmark models have been performed. Superiority over the competing models can genuinely justify the contribution of the predictive structure to the existing strand of cognate literature. Despite the strive toward the accuracy of prediction models, the said frameworks offer very little interpretation owing to black box operational procedure. On the other hand, it is equally important to extract deeper insights into the drivers of financial stress in short and long-run time horizons. To accomplish the endeavor, the underlying work invokes the emerging explainable artificial intelligence (XAI) methodologies to gauge the influence pattern of chosen technical indicators. We have used the permutation feature evaluation scheme to understand the contribution structure of the underlying features globally, while the local level impact of the same is decoded through the lens of the local interpretable model-agnostic explanations (LIME) framework. The implications are specifically critical for traders and investors to track the future figures of financial stress accordingly. Overall, the novelty of the integrated research approach lies in the systematic integration of predictive analysis and model explanation together to precisely forecast the select financial stress indicators in an extremely volatile environment and simultaneously draw insights into the influence of the explanatory features. Thus, the underlying work substantially bridges the research void of financial stress prediction and also imparts contribution to the methodological front.

The remaining section of the article is organized as follows. A summary of the previous cognate literature has been discussed alongside potential research gaps in “Cognate literature”. The section clearly outlines the need for underlying work. Brief descriptions of underlying variables used in this work, along with the key statistical properties, have been outlined in “Variable description”. The said section provides critical insights pertinent to utilized datasets which eventually assist in developing the predictive architectures. The entire research methodology has been thoroughly elucidated subsequently in “Methodology”. Detailed descriptions of individual tools of predictive structure, the workflow of predictive modeling, and utilized performance indicators are enunciated. Next, “Results and analysis” presents the results of predictive performance and thorough analysis. Important implications have been discussed as well in the section. Finally, the paper is concluded in “Conclusion”, highlighting the major findings, implications, and future research directions crisply.

## Cognate literature

Assessment of the nature of financial stress and its influence on other assets are of paramount significance owing to close interactions. This section reviews the previous literature on financial stress and relevant predictive architectures for modeling highly volatile financial assets. It should be noted that process of conceptualization and defining the financial stress index has itself seen a serious attempt in literature. Chadwick and Ozturk [5] utilized principal component analysis (PCA) to construct a financial system stress indicator for Turkey on the basis of money, bond, forex, equity, and bank markets. Ishrakieh et al. [25] developed a financial stress indicator for the Lebanese market comprising separate market sectors: the banking sector, equity, and forex market, and other sectors. On the other hand, OFR financial stress index, too, has been widely accepted as a proxy of stress in five indicators and three regions through embodying 33 financial market variables. Since it is very hard to find notable research work in testing the predictability of financial stress, the section attempts to summarize the recent development of predictive modeling literature on different financial variables.

### Interplay of financial stress with other assets

The work of Ferrer et al. [11] demonstrated that financial stress in the US had a severe impact on the real economy during major financial turmoil and persisted for a longer duration. Das et al. [8] observed strong nexus of global financial stress with gold, crude oil, and stock markets. Empirical research by He et al. [22] demonstrated that financial stress in conjunction with gold price adversely influenced the clean energy stock movements in the US and European markets. In the study by Zhang and Wang [45], in US and China, Bitcoin and gold markets were highly susceptible to financial stress in extremely volatile regimes in short run time horizons. Gkillas et al. [18] demonstrated that financial stress indices significantly explained the variation of oil price volatility and assisted in forecasting the latter. Polat and Ozkan [37] showed how a high quantum of financial stress adversely influenced real economic activities in Turkey. Financial stress, on the other hand, has been found to be driven by oil price shocks [31]. Rho and Saenz [40] found that financial stress was responsible for the spike in the impact of debt, currency reserves, and GDP per capita. Elsayed and Yarovaya [10] showed that instability and turmoil owing to the Arab spring intensified the transmission of financial stress in the MENA region in the short run.

Thus, it can be concluded that financial stress felt across heterogeneous assets and different locations need thorough monitoring to check adversarial effects on critical financial and economic health. On the flip side, to combat the influence of financial stress, precise estimation of futuristic movements of financial stress becomes absolutely vital. Specifically, an attempt should be made to perform predictive analytics at the onset of the COVID-19 pandemic as the level of uncertainty and fear reached a peak during the said time horizon. Unfortunately, to our best knowledge, no such studies have been undertaken either to date. Nexus with heterogeneous financial assets and a dearth of robust predictive structures make the identification of explanatory features to design forecasting frameworks arduous. Therefore, endeavors of underlying research become necessary to fill the existing research void. We now enunciate the trend of predictive modeling frameworks in the context of modeling different financial time series.

### Predictive modeling of financial assets

Henrique et al. [21] conducted a bibliographic review of 57 research articles dedicated to machine learning-based modeling for predictive analysis of financial markets. It was revealed that support vector machine (SVM) and artificial neural network (ANN) were the most commonly used tools. Ghosh et al. [15] combined econometric modeling in conjunction with maximal overlap discrete wavelet transformation (MODWT) and seven machine and deep learning models for constructing granular forecasting frameworks to estimate the one-day ahead movement of stock indices of emerging economies in Asia. Results validated the effectiveness of the presented architectures. Khattak et al. [28] utilized the least absolute shrinkage and selection operator (LASSO) to carry out predictive modeling of the European stock market. The overall findings critically assisted in identifying key predictors efficiently. Mohanty et al. [33] developed a hybrid deep learning-based framework using an autoencoder and kernel extreme learning machine for predictive modeling of high-frequency financial markets. Rigorous performance checks duly rationalized the efficacy of the proposed model. Jana et al. [27] developed a differential evolution (DE) metaheuristic-based integrated framework for short and long-run bitcoin price forecasting wherein maximal overlap discrete wavelet transformation (MODWT) was relied upon for decomposition of original time series into granular components while support vector regression (SVR) and polynomial regression with interactions (PRI) for modeling the governing pattern. Scrupulous performance evaluation demonstrated the efficacy of the proposed model over several benchmark ones. Ghosh and Datta Chaudhuri [16] developed stacking and deep neural network (DNN) driven two distinct frameworks with dedicated frameworks for feature engineering and sorting class imbalance issues for stock trend prediction of the Indian market in pre and post-COVID-19 phases separately. Both modeling structures emerged to be highly successful in the precise estimation of trends in highly volatile periods. There exists a sizeable literature wherein the sentiments of the external floating news and chaotic events have been leveraged to predict stock market trends [2, 9, 30, 32].

A Survey of previous research clearly suggests clear domination of high-end machine and deep learning modeling in constructing forecasting frameworks for financial variables. Deployment of decomposition-driven approaches has prevailed to be pretty effective as well as same assists in separating linear and nonlinear components of highly volatile time series and thereby augmenting the predictive performance. As the financial stress index is naturally expected to exhibit a high degree of volatility and nonlinearity, the underlying work incorporates a decomposition-based granular framework in predicting future figures. We have resorted to the EEMD technique to accomplish the task. Facebook's Prophet is an emerging time series prediction algorithm that has been reported to yield high-quality, superior forecasts of late. Prophet has been reported to be highly robust to outliers, missing data, regime shifts, etc., and successful in complex predictive modeling tasks [1, 35, 44]. It, nevertheless, has not been considerably tested on modeling complex financial time series modeling yet. The work includes Prophet as a predictive modeling tool in conjunction with EEMD to check the predictability of financial stress. LSTM, a well-known and established deep learning tool proven to be extremely efficient in modeling the financial market, has been employed together with EEMD modeling separately for performing predictive analysis. Hence, methodologically the underlying work attempts to contribute to existing literature too.

## Variable description

**Fig. 1:**
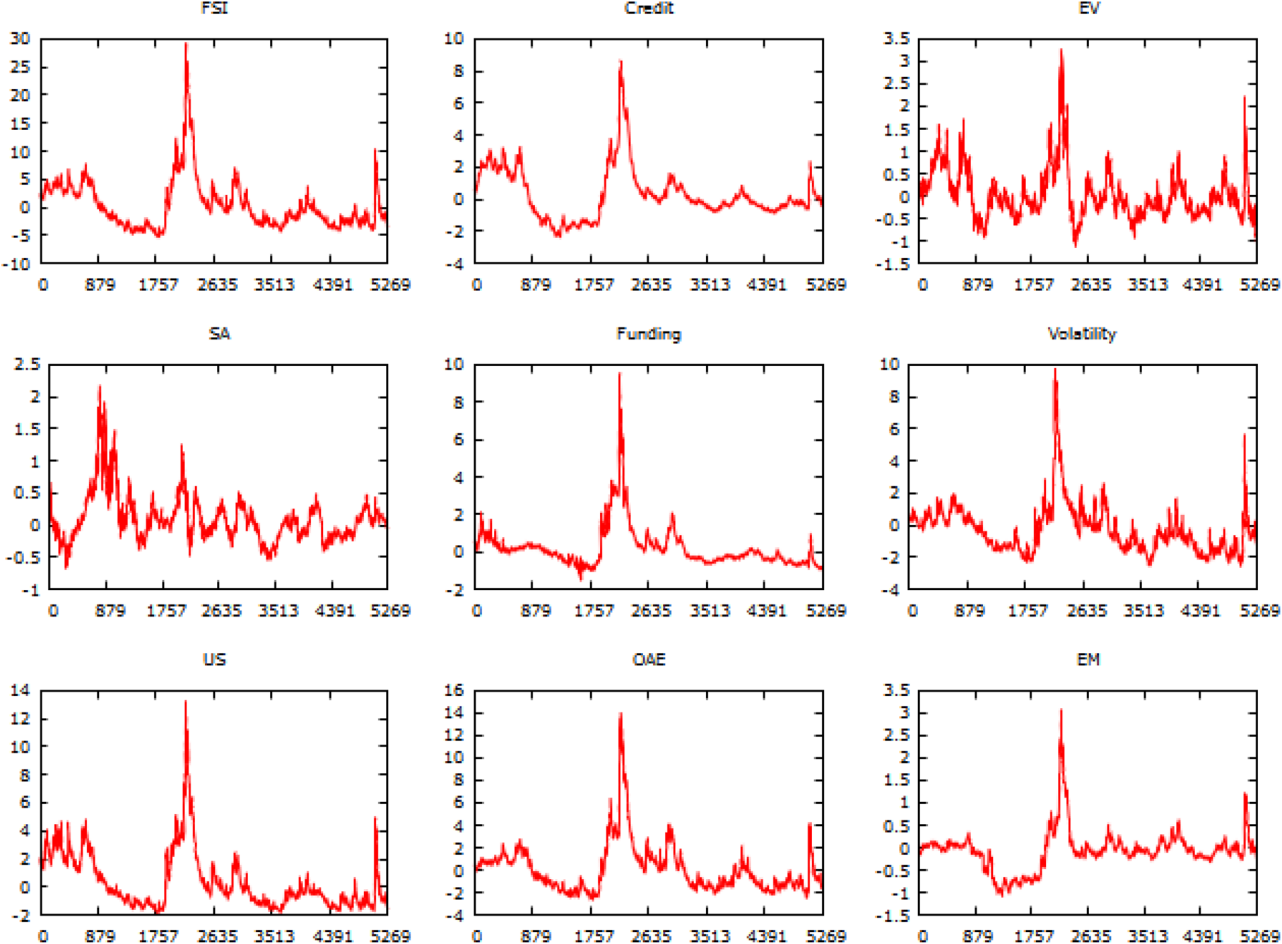
temporal evolutionary pattern of chosen financial stress variables

**Fig. 2:**
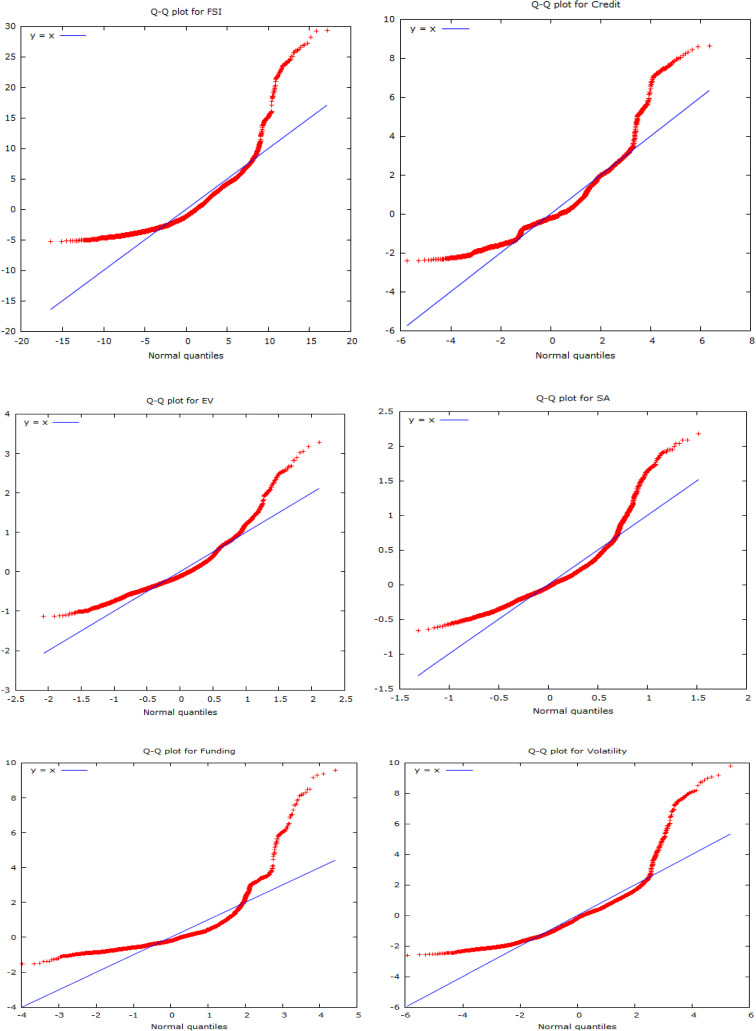

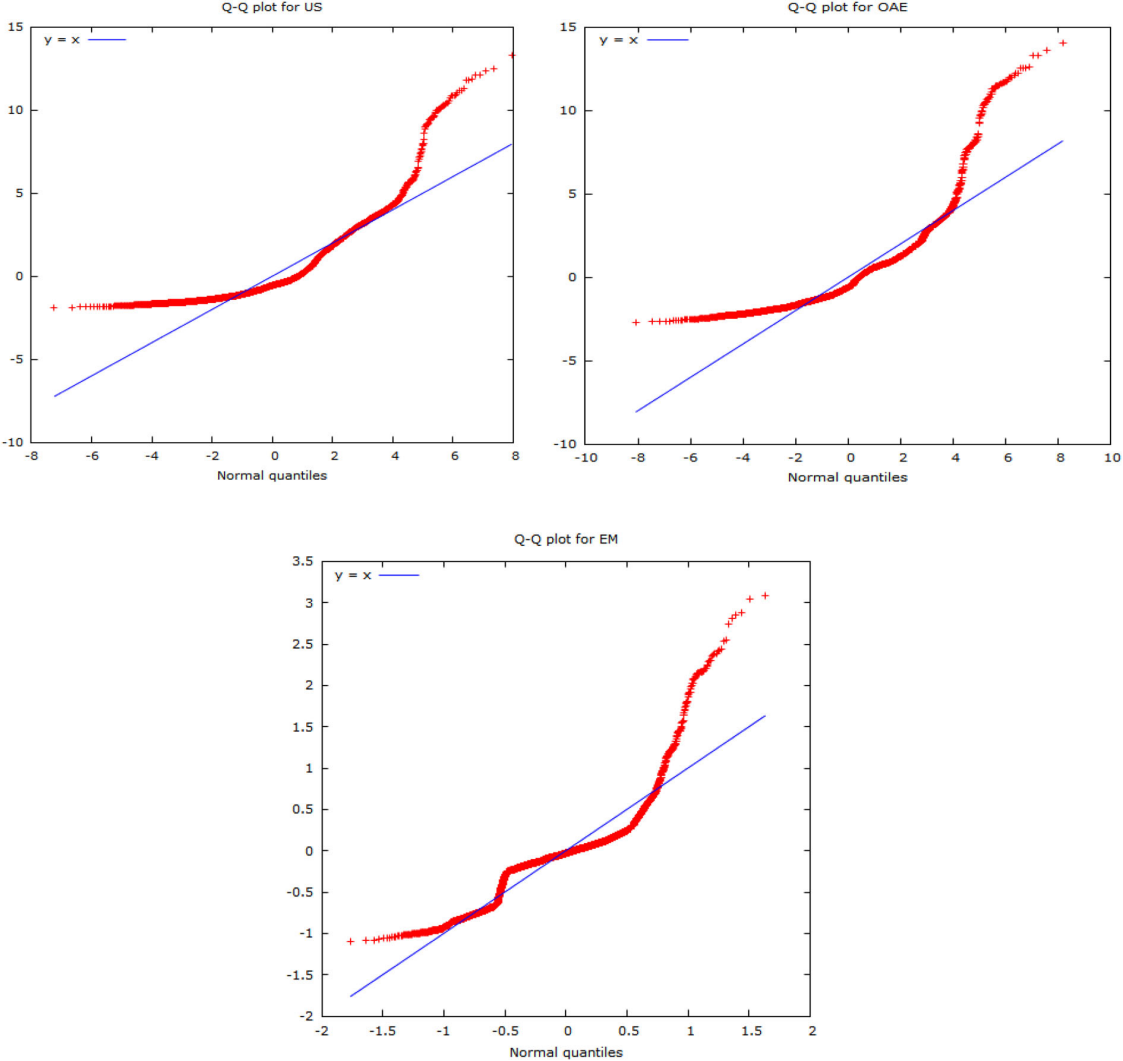
*Q*–*Q* plots of underlying series

Daily observations of FSI, Credit, EV, SA, Funding, Volatility, US, OAE, and EM spanning from January 3, 2000, to December 11, 2020, have been compiled from the official data repository site of OFR (https://www.financialresearch.gov/financial-stress-index/). The FSI measure comprises 33 financial market variables, including yield, spreads, valuation measures, and interest rate. To monitor financial vulnerabilities, OFR evaluates stress across the five categories. Credit accounts for spreads in credit, EV reflects investor confidence and risk appetite, SA monitors the valuation of assets, Funding reflects the flexibility of financial institutions, and Volatility accounts for the implied and realized volatility of equity, credit, currency, and commodity markets. The region-centric variables of stress, US, OAE, and EM account for stress in the US market, Eurozone and Japan markets, and markets of other developing economies respectively. The following exhibit depicts the temporal evolutionary pattern of underlying variables (Figs. 1, 2).

We next amass the descriptive statistics and outcome of several tests in Table 1 to comprehend the fundamental temporal characteristics of underlying variables.

**Table 1:** Summary Statistics Reflecting Key Properties of Underlying Series

Property	FSI	Credit	EV	SA	Funding	Volatility	US	OAE	EM
Minimum	− 5.334	− 2.417	− 1.142	− 0.668	− 1.512	− 2.597	− 1.892	− 2.691	− 1.094
Maximum	29.32	8.62	3.281	2.177	9.582	9.787	13.279	14.057	3.08
Mean	0.3423	0.3037	0.02097	0.1031	0.213	− 0.2985	0.354	0.053	− 0.0652
Median	− 0.877	− 0.070	− 0.103	0.049	− 0.058	− 0.512	− 0.388	− 0.570	− 0.059
Skewness	2.23	1.545	1.393	1.705	3.142	2.068	1.916	2.350	1.496
Kurtosis	7.843	3.920	3.187	4.610	14.319	7.780	5.398	8.345	7.343
Jarque–Bera Test	17,897***	5478***	3938.8***	7226.8***	53,747***	17,065***	9631.2***	20,165***	13,824***
ARCH LM Test	5199.9***	5246.8***	5027.5***	5132***	5207.5***	5102***	5183.2***	5190.7***	5216.3***
Terasvirta’s NN Test	17.746***	1.4417#	21.384***	7.5879**	20.898***	20.611***	10.374***	26.363***	3.7397#
Hurst Exponent	0.8259	0.8258	0.7994	0.8325	0.8471	0.8289	0.8332	0.8455	0.8415

*LM* Autoregressive Conditional Heteroskedasticity Lagrange Multipler, *NN* Neural Network

^#^Not Significant

**Significant 5% Level of Significance

***Significant at 1% Level of Significance, ARCH

Jarque–Bera test statistics indicate the chosen time series observations do not abide by the normal distribution. On the other hand, the significance level of the ARCH LM test suggests the existence of conditional heteroscedasticity entrenched in the evolutionary pattern of underlying variables. The outcome of Terasvirta's NN test implies barring Credit and EM; the remaining six series exhibit a high degree of nonlinearity. Thus, it can be concluded that financial stress series, as anticipated to be highly volatile and uncertain, have eventually appeared to display a strong degree of nonparametric, heteroskedastic, and nonlinear movements. The following Normal *Q*–*Q* plots on selected variables also conform to the findings of the Jarque–Bera test, implying the existence of nonparametric behavior.

The aforesaid plots clearly indicate the presence of skewed distribution, which in turn suggests a violation of normal distribution. The said behavior rationalizes the deployment nonparametric research framework.

On the other hand, figures for the estimated Hurst exponent have been found to be substantially greater than 0.5, which indicates the presence of long memory dependence [14]. Long memory dependence implies that futuristic movement predominantly depends on historical information. Therefore, the usage of technical indicators as explanatory features is duly rationalized. Table 2 summarizes the definition of selected technical indicators as explanatory features in this work.

**Table 2:** Definition of utilized technical indicators

Sl. no.	Features	Formulae
1	One day back closing price (LAG1)	$\mathrm{LAG}1={P}_{i-1}$ where ${P}_{i-1}$ denotes observation of the previous day
2	Two-day back closing price (LAG2)	$\mathrm{LAG}2={P}_{i-2}$
3	Three-day back closing price (LAG3)	$\mathrm{LAG}3={P}_{i-3}$
4	Four-day back closing price (LAG4)	$\mathrm{LAG}4={P}_{i-4}$
5	Five-day back closing price (LAG5)	$\mathrm{LAG}5={P}_{i-5}$
6	5-day moving average (MA5)	$\mathrm{MA}5=\frac{\sum_{i=j-4}^{j}{P}_{i}}{5}$
7	10-day moving average (MA10)	$\mathrm{MA}10=\frac{\sum_{i=j-9}^{j}{P}_{i}}{10}$
8	20-day moving average (MA20)	$\mathrm{MA}20=\frac{\sum_{i=j-19}^{j}{P}_{i}}{20}$
9	5-day bias (B5)	$B5=\frac{{P}_{i}-MA5}{MA5}$
10	10-day bias (B10)	$B10=\frac{{P}_{i}-MA10}{MA10}$
11	20-day bias (B20)	$B20=\frac{{P}_{i}-MA20}{MA20}$
12	5-day momentum (MTM5)	$\mathrm{MTM}5={P}_{i}-{P}_{i-5}$
13	10-day momentum (MTM10)	$\mathrm{MTM}10={P}_{i}-{P}_{i-10}$
14	20-day momentum (MTM20)	$\mathrm{MTM}20={P}_{i}-{P}_{i-20}$
15	5-day rate of change (ROC5)	$\mathrm{ROC}5=\frac{{P}_{i}-{P}_{i-5}}{{P}_{i-5}}$
16	10-day rate of change (ROC10)	$\mathrm{ROC}10=\frac{{P}_{i}-{P}_{i-10}}{{EC}_{i-10}}$
17	20-day rate of change (ROC20)	$\mathrm{ROC}20=\frac{{EC}_{i}-{EC}_{i-20}}{{EC}_{i-20}}$
18	5-day exponential moving average (EMA5)	$\mathrm{EMA}5=\frac{2}{5+1}\times {EC}_{5}+\frac{5-1}{5+1}\times \mathrm{EMA}4$, where $\mathrm{EMA}1={P}_{1}$
19	10-day exponential moving average (EMA10)	$\mathrm{EMA}10=\frac{2}{10+1}\times {EC}_{9}+\frac{10-1}{10+1}\times \mathrm{EMA}9$
20	20-day exponential moving average (EMA20)	$\mathrm{MA}20=\frac{2}{20+1}\times {EC}_{19}+\frac{20-1}{20+1}\times \mathrm{EMA}19$
21	Upper bollinger band (UB)	$\mathrm{UB}=\mathrm{MA}20+(20\times {\sigma }_{20})$ where ${\sigma }_{20}$ denotes the standard deviation of the financial stress of the previous 20 days
22	Lower bollinger band (LB)	$\mathrm{LB}=\mathrm{MA}20-(20\times {\sigma }_{20})$
23	Moving average convergence divergence (MACD)	$\mathrm{MACD}=2\times (\mathrm{DIF}-\mathrm{DEA})$; $\mathrm{DIF}=\mathrm{EMA}12-\mathrm{EMA}26$; $\mathrm{DEA}=\mathrm{EMA}(\mathrm{DIF})$

## Methodology

In detail, this section elucidates the utilized research methodologies for accomplishing the research objective of predictive modeling of financial stress. Components utilized from feature selection to predictive analysis have been discussed sequentially.

### Boruta algorithm

It was introduced by Kursa and Rudnicki [29] for performing feature selection tasks. Boruta is essentially an ensemble-based machine learning algorithm similar to the well-known random forest (RF) tool. It mimics the operational steps with extra ramifications in carrying out the feature selection process. RF, on the contrary, has been reported to suffer from several shortcomings [41]. To tackle the issues, Boruta uses an augmented level of randomness in the existing system for critically expounding the appropriate features. It basically perturbs the underlying features to generate several shadow attributes. Subsequently, the original features are compared against the shadow ones to ascertain the explanatory capabilities of respective features in an ensemble manner. The simulation has been carried out using the *‘Boruta’* library of R software.

### Ensemble empirical mode decomposition (EEMD)

It was originated and developed by Huang et al. [24]. EEMD is a variant of orthodox empirical mode decomposition (EMD), mainly used for decomposing highly nonlinear and non-stationary time series into granular subseries, commonly referred to as intrinsic mode functions (IMFs) and a residual component. The major drawback of traditional EMD, i.e., mode mixing problem, can be overcome using EEMD. The operational steps of EEMD are enunciated below.

*Step 1:* Original time series observations are randomly perturbed by the addition of noise components for creating a noise-added version of the series

1 $${X}^{i}\left(t\right)=X\left(t\right)+\varepsilon \left(t\right), i\in 1,\dots ,I$$

where, $\varepsilon \left(t\right)$ denotes independent Gaussian white noise, and *I* is the number of trials.

*Step 2:* Classical EMD is invoked on transformed series to extract IMFs and residual.

2 $${X}^{i}\left(t\right)=\sum_{j=1}^{N}{C}_{j}^{i}+{r}_{N}^{i}$$

*Step 3:* Average outcome of all trials is estimated to retrieve the original series using the following equation

3 $$X\left(t\right)=\frac{1}{I}\left(\sum_{i=1}^{I}\sum_{j=1}^{N}{C}_{j}^{i}+{r}_{N}^{i}\right)+{\varepsilon }_{I}$$

where $${\varepsilon }_{I}=\frac{\varepsilon }{\sqrt{N}}$$

Average operation is necessary for canceling the impact of uncorrelated white noise while keeping meaningful information. The ‘Rlibeemd’ library of R is leveraged for practical implementation.

### Long short-term memory network (LSTM)

LSTM, conceptualized by Hochreiter and Schmidhuber [23], is basically a modified version of a classical recurrent neural network capable of thwarting well-known *‘vanishing gradient’* problem while performing complex pattern mining tasks [12, 19, 20]. It preserves adjacent and long-term information of evolutionary pattern time series data for recognizing the hidden pattern. The standard LSTM architecture consists of memory cells for keeping records and a set of controlling gates for regulating the flow of information. Memory cells can effectively store short and long-range information as per the needs. Three types of controlling gates exist, namely, input, forget, and output gates for monitoring the flow. Input gates control the amount of present information to be kept in input, while forget gates are responsible for deciding the extent of information to be exchanged with newer ones. Finally, the output gate gets to decide the forward propagation of information through transformations which result in the final output. The input (${I}_{t}$) and cell state (${C}_{t}$) values are governed by the following equations

4 $${I}_{t}=\sigma \left({W}_{i}{x}_{t}+{U}_{i}{h}_{t-1}+{b}_{i}\right)$$

5 $${C}_{t}=\mathrm{tan}h\left({W}_{C}{x}_{t}+{U}_{C}{h}_{t-1}+{b}_{C}\right)$$

where $W, U, b$ represent weight matrices layer wise and bias units while $\sigma$ and $\mathrm{tan}h$ are sigmoid and nonlinear hyperbolic tangent activation functions.

The output of forget gate ($F\left(t\right))$ is estimated as:

6 $$F\left(t\right)=\sigma \left({W}_{f}{x}_{t}+{U}_{f}{h}_{t-1}+{b}_{f}\right)$$

The memory cell state is updated as:

7 $${\widetilde{C}}_{t}={F\left(t\right)I}_{t}\times {C}_{t}+{F}_{t}\times {C}_{t-1}$$

The outcome of output gates is computed as

8 $${O}_{t}= \sigma \left({W}_{o}{x}_{t}+{U}_{o}{h}_{t-1}+{V}_{o}{C}_{t}+{b}_{o}\right)$$

The final output is then estimated as:

9 $${F\_O}_{t}={O}_{t}\times \mathrm{tan}h\left({C}_{t}\right)$$

The present work utilizes the ‘Keras’ framework in the Python programming environment for implementing the LSTM model. Three LSTM blocks of 30 neurons each are considered for modeling. The back-propagation through time (BPTT) has been used as the learning algorithm. Other salient process parameters, viz. Learning rate, batch size, activation functions, etc., are auto-tuned using the *‘GridSearchCV’* utility of Python's *'sklearn’* library.

### Facebook’s prophet

Prophet, developed by Facebook's core data scientists, is an applied predictive modeling algorithm that recently received increased traction in carrying out time series forecasting exercises. It is capable of yielding superior forecasts for complex daily, weekly, monthly, and yearly time series observations through precise segregation of trends, sharp regime shift, seasonality, holiday effects, etc.

The prophet model [43] specification can be expressed as:

10 $$y\left(t\right)=g\left(t\right)+s\left(t\right)+h\left(t\right)+x\left(t\right)+{\epsilon }_{t}$$

where, $y\left(t\right)$ refers to the target construct or time series, $g\left(t\right)$ reflects the trend component accounting for linear or nonlinear effects, $s\left(t\right)$ refers to periodic components, $h\left(t\right)$ measures the holiday effects owing to irregular schedules, and the influence of exogenous features is assessed through $x\left(t\right)$, and finally ${\epsilon }_{t}$ denotes the error term.

For predictive modeling of chosen variables, only the holiday component has not been considered. On the other hand, 24 technical indicators have been chosen as exogenous features for predicting the stress components. Mathematically they can be expressed as:

11 $$\mathrm{FSI}\left(t\right)=g\left(t\right)+s\left(t\right)+\sum\limits_{i=1}^{24}x\left(t\right)+{\epsilon }_{t}$$

12 $$\mathrm{CREDIT}\left(t\right)=g\left(t\right)+s\left(t\right)+\sum\limits_{i=1}^{24}x\left(t\right)+{\epsilon }_{t}$$

13 $$\mathrm{EV}\left(t\right)=g\left(t\right)+s\left(t\right)+\sum\limits_{i=1}^{24}x\left(t\right)+{\epsilon }_{t}$$

14 $$\mathrm{SA}\left(t\right)=g\left(t\right)+s\left(t\right)+\sum\limits_{i=1}^{24}x\left(t\right)+{\epsilon }_{t}$$

15 $$\mathrm{Funding}\left(t\right)=g\left(t\right)+s\left(t\right)+\sum\limits_{i=1}^{24}x\left(t\right)+{\epsilon }_{t}$$

16 $$\mathrm{Volatility}\left(t\right)=g\left(t\right)+s\left(t\right)+\sum\limits_{i=1}^{24}x\left(t\right)+{\epsilon }_{t}$$

17 $$\mathrm{US}\left(t\right)=g\left(t\right)+s\left(t\right)+\sum\limits_{i=1}^{24}x\left(t\right)+{\epsilon }_{t}$$

18 $$\mathrm{OAE}\left(t\right)=g\left(t\right)+s\left(t\right)+\sum\limits_{i=1}^{24}x\left(t\right)+{\epsilon }_{t}$$

19 $$\mathrm{EM}\left(t\right)=g\left(t\right)+s\left(t\right)+\sum\limits_{i=1}^{24}x\left(t\right)+{\epsilon }_{t}$$

We have attempted to model the growth part using piece-wise constant function, which offers high accuracy. Mathematically, it can be expressed as:

20 $$g\left(t\right)=\left(k+a{\left(t\right)}^{T}\delta \right)t+\left(m+a{\left(t\right)}^{T}\gamma \right)$$

Here, $k$ denotes the growth rate, $\delta \left(\in {\mathbb{R}}^{S}\right)$ is the rate adjustment parameter that allows S change points to be incorporated in the model, $m$ denotes the offset parameter, and $\gamma$ controls the magnitude of the rate of change. For daily samples, Prophet automatically estimates weekly and yearly seasonality segments. Seasonality is modeled using a Fourier series as:

21 $$s\left(t\right)=\sum\limits_{n=1}^{N}\left({a}_{n}\mathrm{cos}\left(\frac{2\pi nt}{P}\right)+{b}_{n}\mathrm{sin}\left(\frac{2\pi nt}{P}\right)\right)$$

where $P$ denotes the period of the time series (yearly, weekly, daily, etc.). Therefore modeling seasonality demands computations of $2N$ parameters, $\beta ={\left[{a}_{1}{b}_{1}\dots {a}_{N}{b}_{N}\right]}^{T}$.

The fitting process of the Prophet algorithm applies a maximum posterior probability (MAP) process or full Bayesian statistical inference with Markov Chain Monte Carlo (MCMC) sampling. Once the learning is completed, Prophet can be used for forecasting on the test segment where the average frequency and magnitude of trend change are assumed to be constant. Prophet has emerged to be robust to outliers, missing data, nonlinearity, regime shifts, etc. The emphasis on separately modeling the trend and seasonality components of inherent time series in parallel to incorporating the effects of exogenous variables makes Facebook's Prophet an ideal choice for time series forecasting. The provision of Fourier series-driven seasonality incorporation in varying time horizons is useful for anticipating abrupt changes. The 'fbprophet' library has been used for simulating the model, which additionally provides an automatic change point detection facility too. Due to these advantageous points, Facebook's Prophet has recently seen remarkable success in modeling complex time series variables [17, 26].

### Performance indicators

To evaluate the degree of accuracy of forecasts obtained using LSTM and Prophet, three dedicated measures namely, Nash–Sutcliffe Efficiency ($\mathrm{NSE}$), Index of Agreement ($\mathrm{IA}$), and Theil’s Inequality Coefficient ($\mathrm{TI}$) have been utilized. Mathematically they are computed as:

22 $$\mathrm{NSE}=1-\frac{\sum_{i=1}^{N}{\left\{{\mathrm{Y}}_{\mathrm{act}}\left(\mathrm{i}\right)-{\mathrm{Y}}_{\mathrm{pred}}\left(\mathrm{i}\right)\right\}}^{2}}{\sum_{i=1}^{N}{\left\{{\mathrm{Y}}_{\mathrm{act}}\left(\mathrm{i}\right)-\overline{{Y }_{act}}\right\}}^{2}}$$

23 $$\mathrm{IA}=1-\frac{\sum_{i=1}^{N}{\left\{{Y}_{act}\left(i\right)-{Y}_{pred}(i)\right\}}^{2}}{\sum_{i=1}^{N}{\left\{\left|{Y}_{pred}\left(i\right)-\overline{{Y }_{act}}\right|+\left|{Y}_{act}\left(i\right)-\overline{{Y }_{act}}\right|\right\}}^{2}}$$

24 $$\mathrm{TI}=\frac{{\left[\frac{1}{N}\sum_{i=1}^{N}{\left({\mathrm{Y}}_{\mathrm{act}}\left(\mathrm{i}\right)-{\mathrm{Y}}_{\mathrm{pred}}\left(\mathrm{i}\right)\right)}^{2}\right]}^{1/2}}{{\left[\frac{1}{N}\sum_{i=1}^{N}{{\mathrm{Y}}_{\mathrm{act}}\left(\mathrm{i}\right)}^{2}\right]}^{1/2}+{\left[\frac{1}{N}\sum_{i=1}^{N}{{\mathrm{Y}}_{\mathrm{pred}}\left(\mathrm{i}\right)}^{2}\right]}^{1/2}}$$

**Fig. 3:**
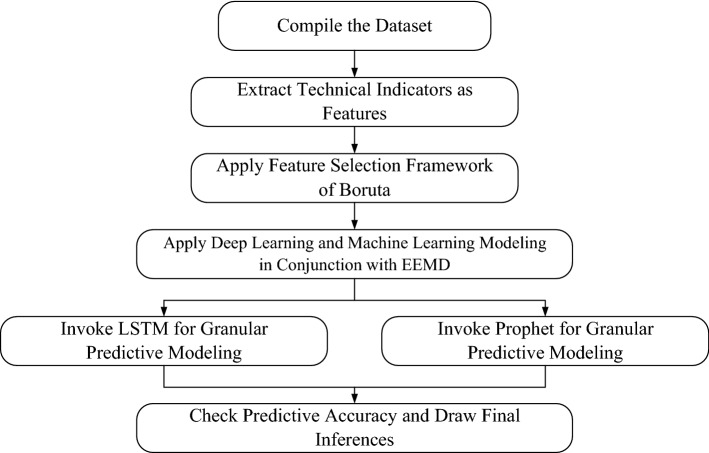
Flowchart of the predictive framework

The range of NSE lies between—∞ to 1. Values close to 1 suggest superior forecasts, while values less than 0 signify the model is poor than the observed mean-based prediction. Likewise, NSE and IA figures should be high and close to 1 as well for classifying forecasts of supreme accuracy. It ranges from 0 to 1. Lastly, the range of TI Values lies from 0 to 1. Predictions are marked to be of high quality when TI values emerge to close to 0. The following Fig. 3 exhibits a flowchart of the entire research framework.

### Explainable artificial intelligence (XAI)

As elicited earlier, both predictive frameworks, EEMD-LSTM and EEMD-Prophet, are designed to fetch superior predictions at the expense of model interpretation. To understand how the select technical indicators, drive the financial stress in chosen indicators, two dedicated XAI techniques have been utilized to draw insights on global and local scales.

#### Permutation feature importance

Breiman Breiman [4] originally proposed the framework to gauge the influence of explanatory features in predicting the target using the permutation feature importance methodology. Later, Fisher et al. [13] incorporated minor modifications to the original permutation feature importance model to leverage the same as a model agnostic mechanism. In the revised methodological framework, the extent of the impact of any feature is determined by randomly altering its original values to evaluate the impact on the overall predictive accuracy of the model. A higher error implies a stronger influence of the features. Basically, it accounts for the influence of the underlying features at the global scale.

#### Local interpretable model-agnostic explanations (LIME)

Propounded by Ribiero et al. [39], LIME is an emerging XAI methodology tailor-made to uncover machine learning models at a local level. LIME creates a new dataset on top of a learned model by changing the values of the input variables and fetching the predictions of the target variable. Subsequently, it attempts to interpret the relationship between the target and input variables on the new dataset using relatively better interpretable machine learning models, viz., decision trees, LASSO, etc.

The *‘iml’* package of R has been used for simulating the entire XAI framework.

## Results and analysis

In this section, we discuss the findings of utilized research frameworks to infer the nature of predictability of 9 financial stress series. As stated before, a dedicated feature selection framework using the Boruta algorithm has been used in this work to justify the usage of technical indicators as explanatory features. The framework is capable of identifying irrelevant features as well. Subsequently, decomposition through EEMD and predictive modeling through LSTM and Facebook's Prophet algorithm has been executed. The outcome of the feature selection process is reported initially.

### Outcome of Boruta algorithm

**Fig. 4:**
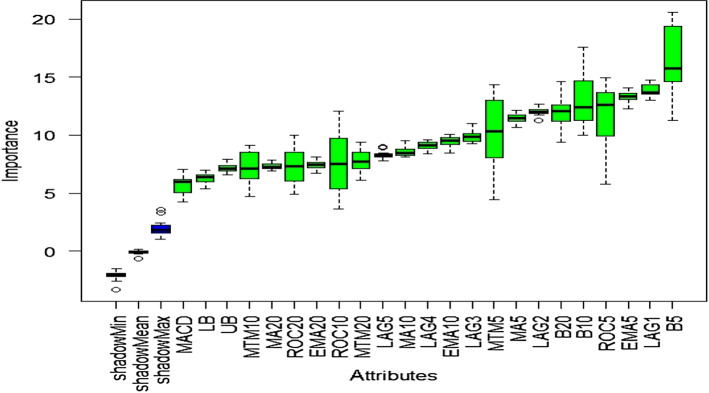
The outcome of Boruta for FSI

**Fig. 5:**
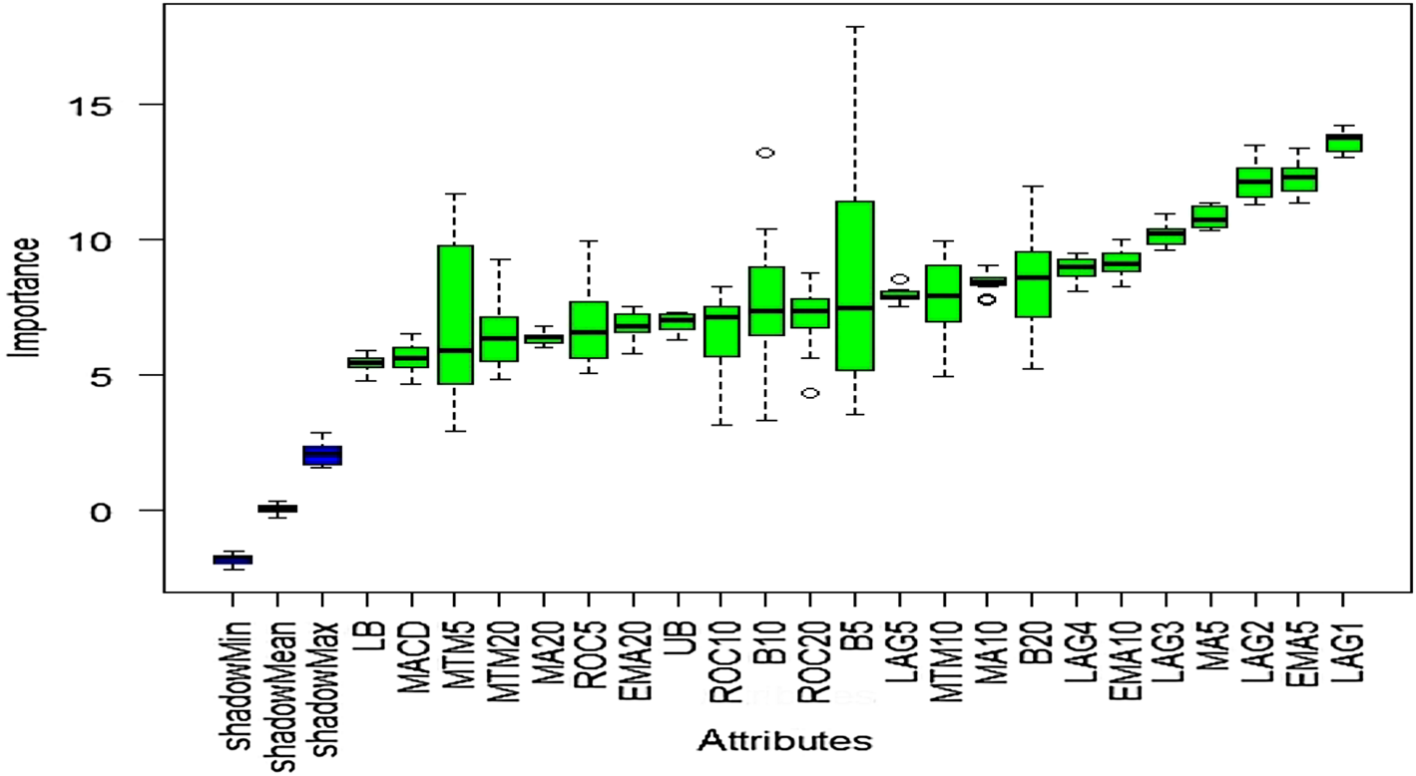
The outcome of Boruta for Credit

**Fig. 6:**
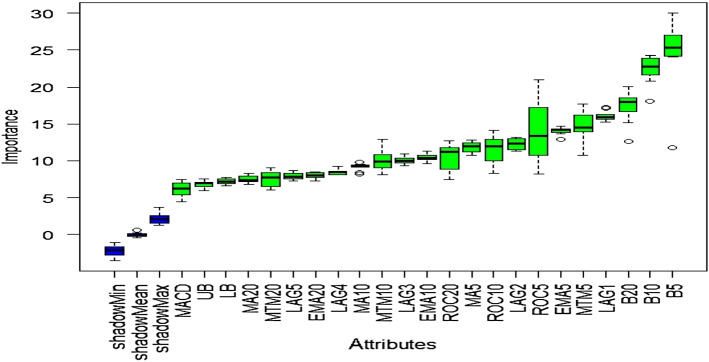
Outcome of Boruta for EV

**Fig. 7:**
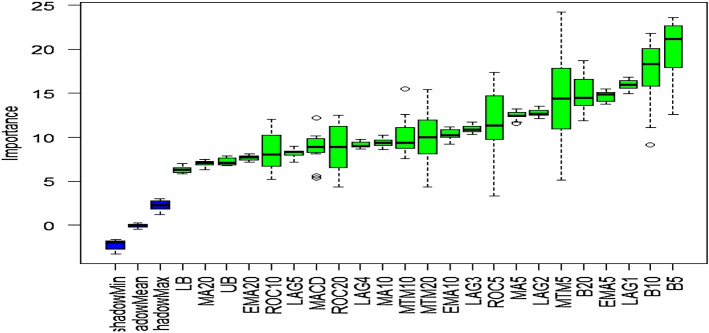
The outcome of Boruta for SA

**Fig. 8:**
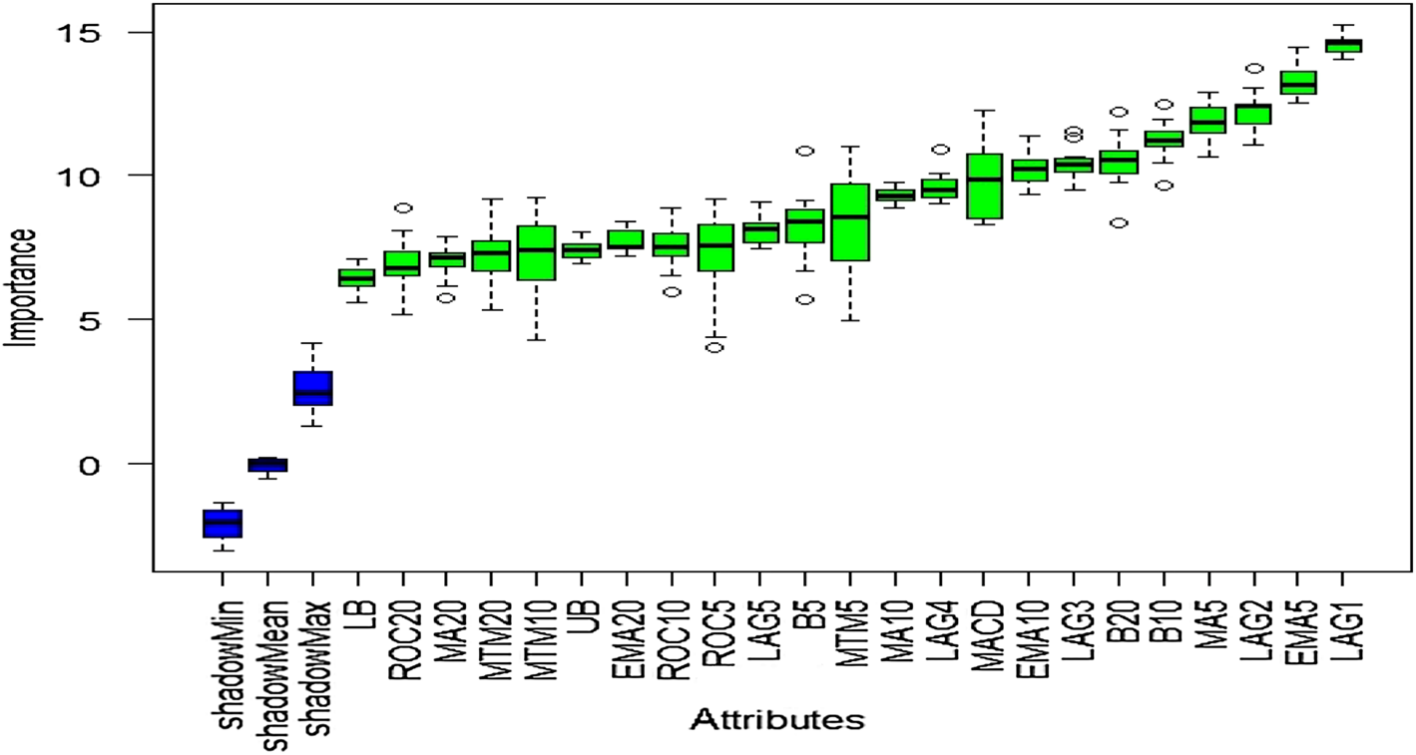
The outcome of Boruta for funding

**Fig. 9:**
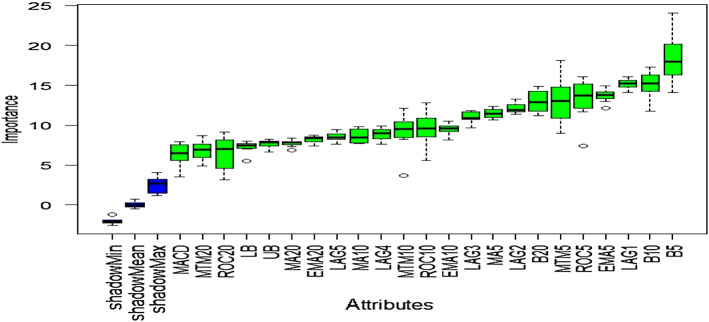
Outcome of Boruta for volatility

**Fig. 10:**
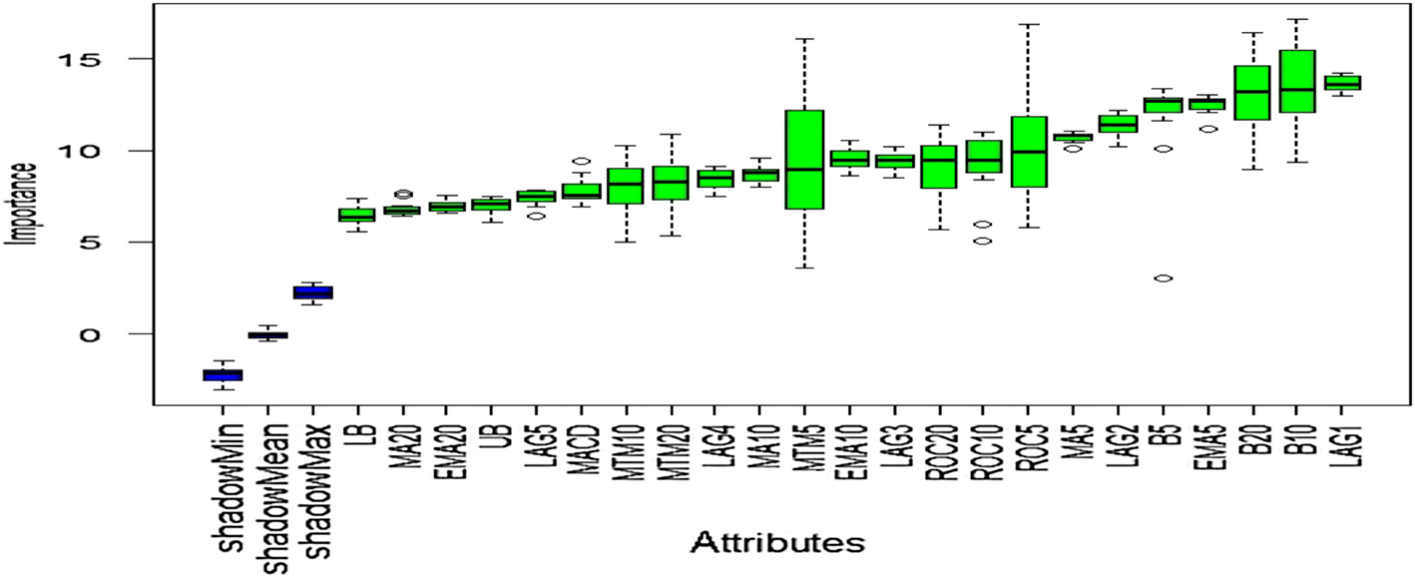
The outcome of Boruta for US

**Fig. 11:**
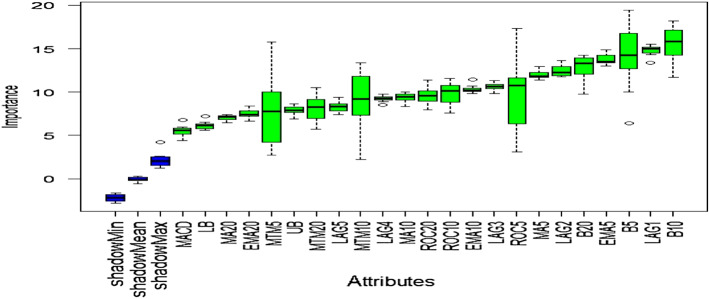
The outcome of Boruta for OAE

**Fig. 12:**
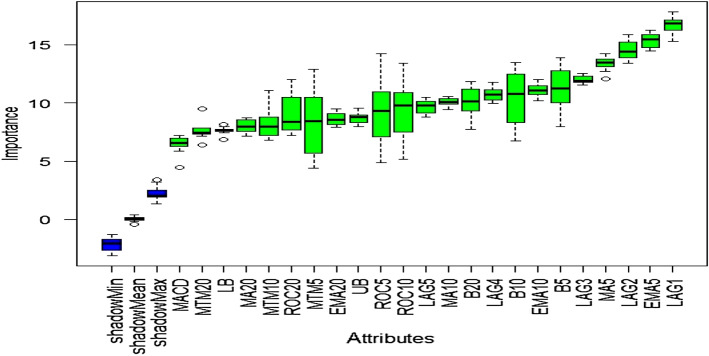
The outcome of Boruta for EM

All 23 technical indicators defined in Table 2 have been subjected to the Boruta algorithm for ascertaining the explanatory power. The outcome of the feature selection process has been visually represented by the following Figs. 4, 5, 6, 7, 8, 9, 10, 11, 12. The figures depict box plots representing the importance scores of respective features obtained through the Boruta algorithm. Features marked in green are inferred to carry significant explanatory capability having significantly better predictability power in comparison to the shadow features marked in blue. Features marked in other colors need further evaluation. It has been revealed that all 23 technical indicators for the time series under consideration have emerged to be significant as the considered features have been found to possess better explanatory capability than the shadow ones. As no features are found in red, the absence of insignificant features can be inferred. Hence, it is not required to discard any features, and choice of relying upon technical indicators is truly defensible.

The outcome of Boruta's inspection for evaluating the explanatory capabilities of technical indicators of FSI suggests they possess significant prediction power as only green boxes have emerged, barring the shadow ones.

Likewise, FSI, technical indicators computed for predicting Credit have turned out to possess significant explanatory capability as none of the attributes are weaker than the shadow ones. Hence, all these features can be effectively leveraged in building granular EEMD-LSTM and EEMD-Prophet frameworks.

Similar to earlier instances, technical indicators computed for inspecting the predictability of EVs have emerged as highly significant. Therefore, all of them can be relied upon for estimating precise forecasts.

The absence of insignificant technical indicators for predictive analysis of SA is amply apparent as well. The outcome of Boruta exhibits the presence of significant explanatory features with substantially higher predictive capability than the shadow attributes.

Visualization of feature evaluation of Funding series through Boruta clearly indicates all underlying technical indicators can be effectively utilized for predictive modeling exercise. None of the features has emerged to be insignificant.

Similar to earlier ones, technical indicators estimated for predicting Volatility series have also appeared to be highly significant, as the plot contains only green and blue boxes reflecting significant and shadow attributes.

All technical indicators of US series have emerged to be statistically significant in terms of predictive power than the shadow features. Therefore, they can be seamlessly deployed to granular forecasting frameworks subsequently.

The predictability of OAE can effectively be checked through the estimated technical indicators as the outcome of Boruta clearly indicates the existence of significant predictive capabilities entrenched in the chosen features.

The outcome of Boruta for the last variable, EM, also implies all presence of significant explanatory power in considered technical indicators.

The above Figs. 4, 5, 6, 7, 8, 9, 10, 11, 12 only comprise green and blue boxes representing the significant and shadow attributes. Thus, the selection of technical indicators as the explanatory feature has emerged to be highly effective. Although all chosen explanatory features have appeared to be significant, it is a good practice to perform a dedicated feature evaluation before proceeding with predictive modeling. Boruta offers a simple yet effective filtering operation as manifested by the outcome.

### Decomposition by EEMD

**Fig. 13:**
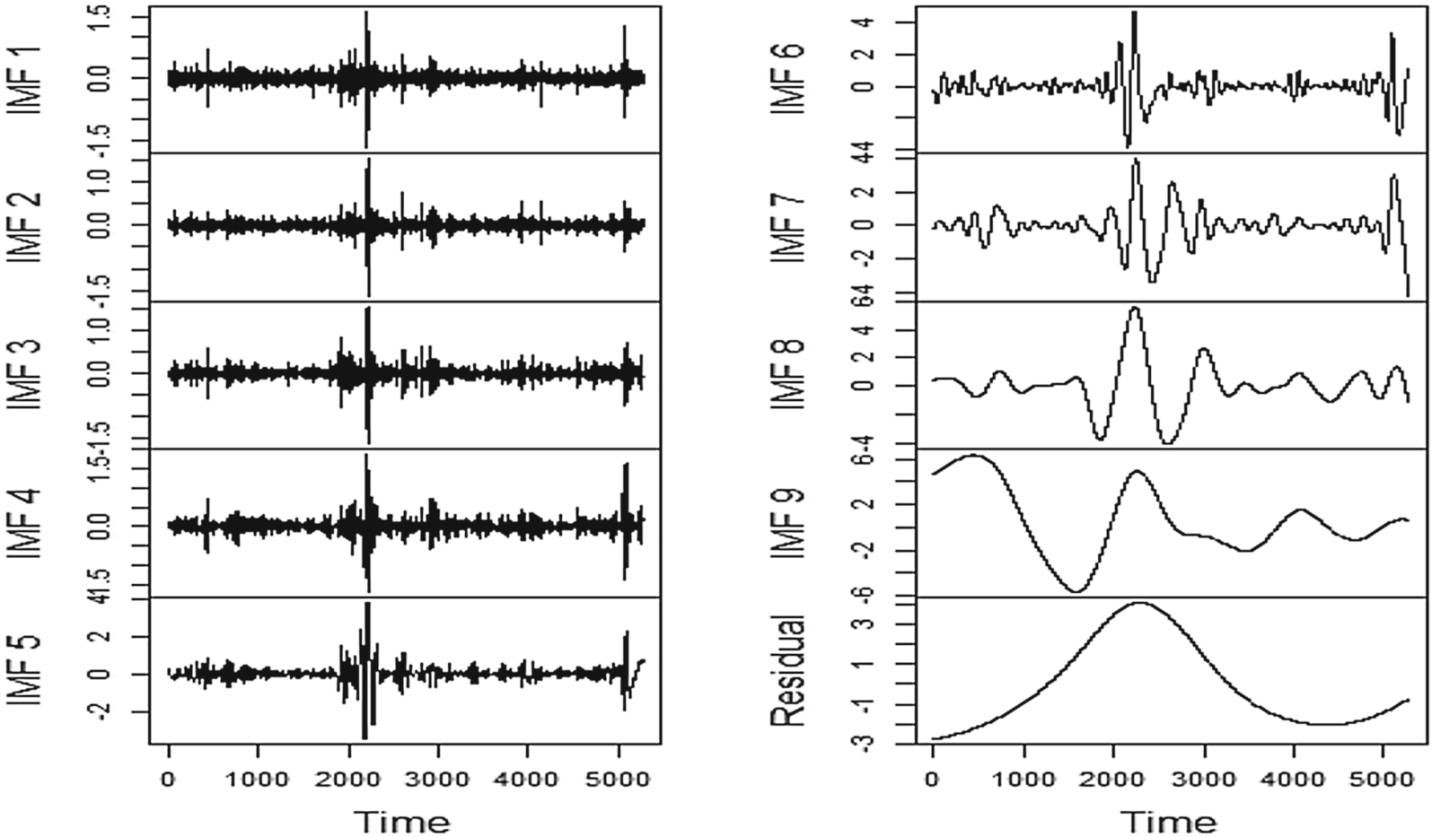
EEMD Decomposition of FSI

**Fig. 14:**
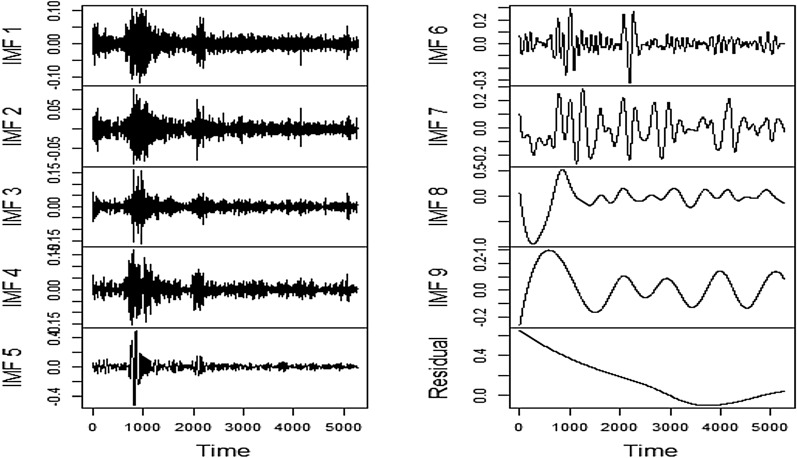
EEMD Decomposition of SA

**Fig. 15:**
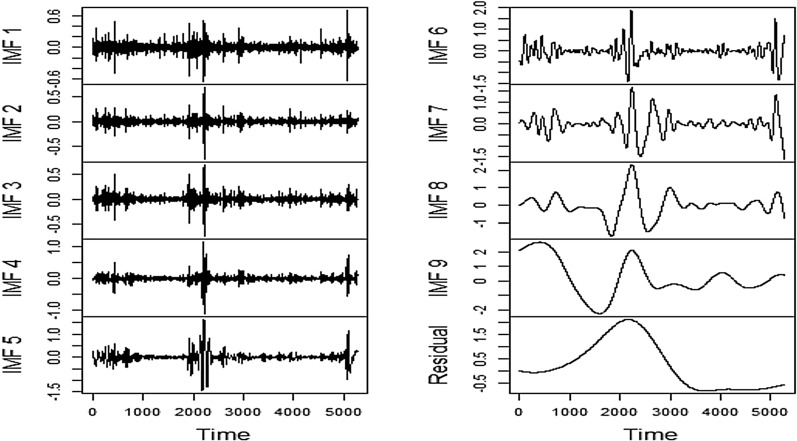
EEMD Decomposition of US

**Fig. 16:**
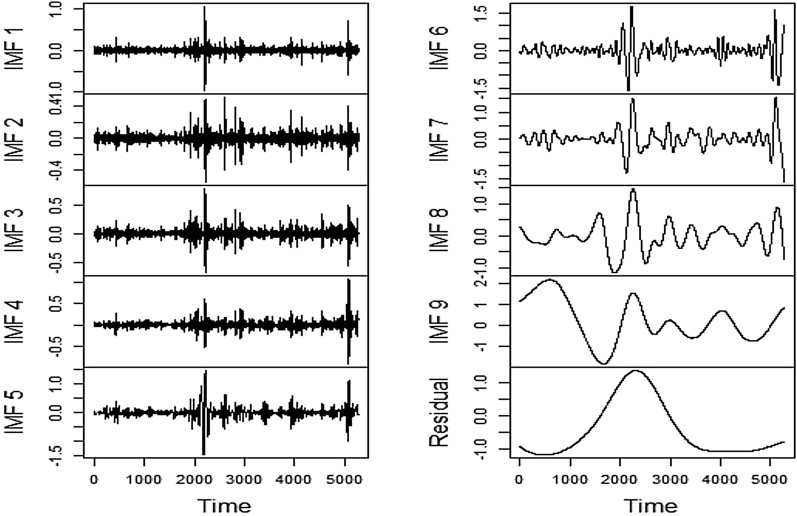
EEMD Decomposition of Volatility

Completion of the feature evaluation process is succeeded by granular decomposition of underlying time series reflecting financial stress using EEMD. Unlike other decomposition algorithms, viz. discrete wavelet transformation, maximal overlap discrete wavelet transformation, etc., the number of subseries, i.e., IMFs to be generated, needs not to be fixed beforehand. The decomposition is carried out until the residual components of the respective series can be found to possess less than two local extrema. In this research, it has been observed that several series needed nine levels of decomposition while others required more than that. The chosen explanatory features have decomposed accordingly as well for setting input and output combinations. For visualization, we have presented the series' decomposition process, which needed nine levels. Figures 13, 14, 15, 16 depict the original and decomposed subseries.

The decomposition plot indicates the dominance of high frequency and nonlinear components in the aggregate FSI series, which would be extremely difficult to predict. However, the decomposition has efficiently disentangled linear and nonlinear parts. Hence from the pattern recognition perspective, the role of EEMD is of paramount importance.

Similar to FSI, the strong presence of a nonlinear component in SA is evident. It can be seen that the high-frequency parts manifested in IMF1, IMF2, IMF3, and IMF4 have emerged to be more intense than the FSI. Hence, the deployment of EEMD in conjunction with LSTM and Prophet is justified once more.

Similar to FSI and SA series, the decomposition of the US series using EEMD has successfully segregated the complex patterns from the relatively easier ones, which in turn assists the subsequent modeling through LSTM and Prophet frameworks immensely.

It is pretty apparent that EEMD has conveniently separated the complex components of the Volatility series as well likewise the earlier cases. Delving predictability, therefore, becomes relatively easier through the lens of predictive modeling.

It can clearly be observed that the decomposition process of EEMD has been quite effective in disentangling the nonlinear and volatile components. The said process can definitely assist LSTM and Prophet in recognizing the apparent complex pattern of stress series with a superior degree of precision. The outcome of the final predictive exercise is discussed next.

### Outcome of predictive modeling

As stated in the introduction, the evaluation of predictability has been conducted through performing static and dynamic forecasting exercises. Static forecasting checks one-step (one-day) ahead prediction quality, whereas dynamic forecasting checks multistep (multiple days) ahead forecasting. Therefore, the extent of predictability of financial stress in short and long-run time horizons can be expounded. Static forecasting evaluation is checked through the deployment of a rolling window in a forward-looking manner across the entire sample. A rolling window of 720 observations has been considered in this work. Average performance on twenty trials has been estimated to infer the quality of static forecasting. To perform dynamic forecasting, observations from January 3, 2000, to December 31, 2017, of all nine series have been used for comprising training samples, while observations pertinent to the remaining period constitute test segments. The test segment also contains a timeline heavily affected by the COVID-19 pandemic. Learning of both LSTM and Prophet frameworks has been accomplished utilizing the training samples and subsequently evaluated on test segments. Performance indicators discussed in “Performance indicators” have been estimated to judge the quality of derived predictions. LSTM has been implemented in *‘Keras’* platform using 'Python' programming framework using the back-propagation through time (BPTT) algorithm for learning. Two hidden layers comprising 50 nodes each have been used. On the other hand, *'*fbprophet' library has been used in the Python programming environment for implementing Facebook's Prophet algorithm. Additive seasonality components using daily periodic patterns and Fourier orders of 20 have been selected for simulating the model. Default figures of the remaining parameters have been used, which are auto-tuned by the method for the best fit. Initially, the performance of static forecasting is reported. Tables 3 and 4 summarize the predictive performance of respective models.

**Table 3:** Performance of EEMD-LSTM modeling in static forecasting

	NSE	TI	IA
Training dataset			
FSI	0.99672	0.00068	0.99759
Credit	0.99784	0.00054	0.99836
EV	0.99741	0.00074	0.99790
SA	0.99813	0.00046	0.99884
Funding	0.99904	0.00049	0.99935
Volatility	0.99587	0.00079	0.99631
US	0.99648	0.00061	0.99698
OAE	0.99926	0.00040	0.99943
EM	0.99939	0.00038	0.99958
Test dataset			
FSI	0.99565	0.00083	0.99633
Credit	0.99693	0.00067	0.99744
EV	0.99617	0.00089	0.99681
SA	0.99740	0.00060	0.99757
Funding	0.99835	0.00065	0.99866
Volatility	0.99442	0.00094	0.99540
US	0.99559	0.00073	0.99604
OAE	0.99862	0.00049	0.99881
EM	0.99894	0.00047	0.99902

**Table 4:** Performance of EEMD-prophet modeling in static forecasting

	NSE	TI	IA
Training dataset			
FSI	0.99663	0.00065	0.99743
Credit	0.99781	0.00056	0.99829
EV	0.99745	0.00077	0.99796
SA	0.99816	0.00049	0.99891
Funding	0.99901	0.00053	0.99929
Volatility	0.99596	0.00076	0.99637
US	0.99634	0.00058	0.99706
OAE	0.99919	0.00042	0.99931
EM	0.99933	0.00041	0.99955
Test dataset			
FSI	0.99557	0.00081	0.99626
Credit	0.99696	0.00069	0.99734
EV	0.99637	0.00092	0.99681
SA	0.99734	0.00063	0.99785
Funding	0.99845	0.00065	0.99957
Volatility	0.99517	0.00086	0.99562
US	0.99565	0.00070	0.99623
OAE	0.99857	0.00054	0.99884
EM	0.99878	0.00051	0.99895

It can be clearly observed that the performance of both EEMD-LSTM and EEMD-Prophet has appeared to be extremely satisfactory on both training and test segments of all financial stress indices. The magnitude of NSE and IA has emerged to be pretty high, very close to 1 on both training and test cases. On the other hand, TI figures have been found to be quite low as well. Thus, inference can be drawn that the LSTM and Prophet have successfully captured the inherent pattern underlying time series in conjunction with the decomposition framework of EEMD. Hence, the superior performance of static forecasting clearly suggests that one-day ahead, future figures of underlying financial stress indices can indeed be predicted with utmost efficiency. Therefore, the future trend of fear, uncertainty, volatility, etc. can be estimated. Precise prediction of a one-day ahead quantum of financial stress arising in markets can be exploited for critical practical implications. Next, we examine the performance of both approaches on dynamic setups. Tables 5 and 6 outline the figures for performance indicators in training and test setups.

**Table 5:** Performance of EEMD-LSTM modeling in dynamic forecasting

	NSE	TI	IA
Training dataset			
FSI	0.99113	0.00084	0.99194
Credit	0.99185	0.00063	0.99226
EV	0.99156	0.00082	0.99201
SA	0.99214	0.00060	0.99247
Funding	0.99281	0.00065	0.99258
Volatility	0.98996	0.00092	0.99126
US	0.99107	0.00079	0.99151
OAE	0.99337	0.00057	0.99388
EM	0.99308	0.00054	0.99370
Test dataset			
FSI	0.98935	0.00102	0.99023
Credit	0.98994	0.00084	0.99074
EV	0.98965	0.00105	0.99045
SA	0.99013	0.00079	0.99089
Funding	0.99095	0.00088	0.99101
Volatility	0.98813	0.00124	0.98912
US	0.98904	0.00097	0.98997
OAE	0.99167	0.00080	0.99185
EM	0.99144	0.00077	0.99156

**Table 6:** Performance of EEMD-Prophet Modeling in Dynamic Forecasting

	NSE	TI	IA
Training dataset			
FSI	0.98996	0.00088	0.99180
Credit	0.99175	0.00065	0.99219
EV	0.99141	0.00083	0.99193
SA	0.99189	0.00061	0.99244
Funding	0.99277	0.00067	0.99256
Volatility	0.98989	0.00092	0.99111
US	0.99109	0.00083	0.99142
OAE	0.99328	0.00058	0.99379
EM	0.99304	0.00056	0.99365
Test dataset			
FSI	0.98917	0.00107	0.99007
Credit	0.98989	0.00091	0.99060
EV	0.98962	0.00106	0.99038
SA	0.99002	0.00085	0.99081
Funding	0.99082	0.00087	0.99104
Volatility	0.98817	0.00129	0.98895
US	0.98886	0.00107	0.98993
OAE	0.99155	0.00083	0.99179
EM	0.99132	0.00078	0.99142

It can clearly be observed that likewise static forecasting exercise, both EEMD-LSTM and EEMD-Prophet have emerged to be successful in precisely estimating the future figures of all nine stress-related series in long run duration as manifested by high NSE and IA values, and low TI figures on training and test samples. It can be observed that the figures for performance indicators have marginally deteriorated in comparison to the static forecasting exercise. It must be noted that the test segment of dynamic forecasting includes observations arising during the COVID-19 pandemic. Thus, it is quite natural to experience a dip in performance as dynamic forecasting is a tad difficult too. Nevertheless, an inference can be drawn that financial stress can be effectively forecasted in the long run.

In a nutshell, evidence from static and dynamic prediction exercises duly rationalizes the efficacy of both EEMD-LSTM and EEMD-Prophet in decoding the temporal evolution pattern of financial stress reflected in 9 categories. Financial stress is indeed predictable based on the historical information of computed technical indicators. Accuracy of prediction is pretty high in one-day ahead forecasting while the quality of predictions in multi-day ahead setup is above satisfactory level as well. Hence, it is possible to anticipate stress in the financial market beforehand, which can be leveraged in mitigating risk in the form of alienating the detrimental impacts on economic and financial health through policy implementation, strategic intervention, etc. Facebook's Prophet, which has been regarded to be extremely promising in mining complex patterns, has emerged to be pretty successful in modeling highly volatile financial time series, while the deployment of technical indicators turns out to be effective also. Therefore, the underlying research emerges to contribute from methodological aspects as well.

We have additionally performed a model confidence set (MCS) based superior predictability assessment to relatively rank the underlying nine financial stress indicators based on the degree of predictability. MCS modeling is capable of discriminating the superior and inferior prediction outcomes through the utilization of several loss functions. Findings of MCS evaluation are presented in following Tables 7 and 8.

**Table 7:** The outcome of MCS evaluation for static forecasting

Model	FSI	Credit	EV	SA	Funding	Volatility	US	OAE	EM
EEMD-LSTM	(7)	(5)	(6)	(4)	(3)	(9)	(8)	(2)	(1)
EEMD-Prophet	(7)	(5)	(6)	(4)	(3)	(9)	(8)	(2)	(1)

**Table 8:** The outcome of MCS evaluation for dynamic forecasting

Model	FSI	Credit	EV	SA	Funding	Volatility	US	OAE	EM
EEMD-LSTM	(7)	(5)	(6)	(4)	(3)	(9)	(8)	(1)	(2)
EEMD-Prophet	(7)	(5)	(6)	(4)	(3)	(9)	(8)	(1)	(2)

It can be seen that under a static forecasting scheme, financial stress in EM and OAE has appeared to be the top two predictable indices, whilst stress in US and volatility have emerged to be the least predictable. It basically implies that the temporal patterns of financial stress reflected in market volatility and US context are relatively highly volatile than their counterparts. Rankings remain uniform across EEMD-LSTM and EEMD-Prophet, both of which basically confirm the homogeneity of predictive performance. Under a dynamic forecasting setup, similar findings can be seen. The only change in outcome is manifested by swapped rankings of EM and OAE. However, the rankings of other series remain constant. Therefore, it can definitely be concluded that the predictability of financial stress varies across different categories.

### Validation and statistical comparative analysis

Although the predictive structures transpire to be highly effective in precisely estimating the future figures of the considered series, it is important to validate the efficiency of both EEMD-LSTM and EEMD-Prophet models in extremely volatile circumstances. Two different experiments have been conducted to meet the requirement. In one setup, the predictability of the chosen variables explicitly during the pandemic timeline, i.e., from April 1, 2020, to June 30, 2022, is delved into, whereas the accuracy of forecasts in surrogate time series of high chaotic traces is tested in the other setup. As static forecasting is relatively easier, we have focused on dynamic prediction exercises for a fair evaluation. Tables 9 and 10 report the predictive analysis outcome on the test data segment.

**Table 9:** Dynamic forecasting performance on test segment in COVID-19 horizon

	NSE	TI	IA
EEMD-LSTM			
FSI	0.97672	0.00376	0.97895
Credit	0.97890	0.00321	0.98071
EV	0.97765	0.00403	0.97924
SA	0.98112	0.00258	0.98265
Funding	0.98236	0.00226	0.98315
Volatility	0.97534	0.00447	0.97803
US	0.97522	0.00474	0.97748
OAE	0.98325	0.00271	0.98446
EM	0.98227	0.00209	0.99308
EEMD-Prophet			
FSI	0.97665	0.00391	0.97861
Credit	0.97878	0.00384	0.98059
EV	0.97753	0.00428	0.97884
SA	0.98081	0.00252	0.98235
Funding	0.98249	0.00237	0.98308
Volatility	0.97518	0.00415	0.97792
US	0.97546	0.00502	0.97725
OAE	0.98307	0.00283	0.98404
EM	0.98216	0.00222	0.99272

**Table 10:** Dynamic forecasting performance on test segment in surrogate series

	NSE	TI	IA
EEMD-LSTM			
FSI	0.96431	0.00535	0.96612
Credit	0.95898	0.00688	0.96135
EV	0.96133	0.00610	0.96198
SA	0.96260	0.00662	0.96376
Funding	0.95892	0.00697	0.96071
Volatility	0.96388	0.00575	0.96524
US	0.95981	0.00646	0.96186
OAE	0.96226	0.00561	0.96289
EM	0.96125	0.00605	0.96189
EEMD-Prophet			
FSI	0.96129	0.00621	0.96339
Credit	0.95784	0.00740	0.96845
EV	0.95921	0.00663	0.96121
SA	0.96134	0.00736	0.96227
Funding	0.95713	0.00778	0.95833
Volatility	0.96198	0.00685	0.95902
US	0.96204	0.00629	0.96386
OAE	0.96178	0.00606	0.96215
EM	0.96193	0.00587	0.96268

It can be seen that the performance of both models transpires to be above satisfactory level, as manifested by the three performance indicators. It is evident that the performance of the respective models in the test segment has experienced a marginal dip compared to the original forecasting exercises on the aggregate data. However, considering the time horizon of the study, when a possibility of a severe market crash was anticipated, the extent of accuracy is encouraging.

We utilize the 'tseriesEntropy' library of the R platform to generate the surrogate series characterized by high fluctuations leveraging the Sieve Bootsrap (Buhlmann, 1997) procedure. The surrogate series are generated for individual financial stress indicators considering the original sample of observations. The resultant series are more chaotic and volatile compared to the original counterparts. Therefore, if the models EEMD-LSTM and EEMD-Prophet emerge to produce satisfactory accuracy in drawing forecasts, their efficiency in predicting highly chaotic time series will be established.

It is apparent that the performance of the respective models on accurately predicting the surrogate series is satisfactory as the values of both NSE and IA are above greater than 0.95, while TI has remained to be substantially low as well. A close inspection of the estimated figures of the performance indicators reveals that similar to explicit predictive exercise during the COVID-19 period, the precision of predictions is not as good as that of the aggregate data. Nonetheless, the dip in accuracy is expected as the surrogate data series embody a high degree of chaotic and random fluctuations.

We next proceed to a comparative evaluation of both frameworks. We have performed a comparative statistical analysis considering four well-known competing models. The comparison encompasses two univariate econometric forecasting techniques, namely, autoregressive integrated moving average (ARIMA) and seasonal ARIMA (SARIMA), which offer comparatively more interpretability and are faster. We also use a conventional one hidden layer-based multi-layer perceptron neural network (MLP) with 20 hidden nodes for fetching forecasts. The comparison includes robust Bayesian structural time series forecasting framework as well. The BSTSF [42] amalgamates structural time series frameworks by applying Bayesian statistics and fundamental state-space representations. The BSTSF approach performs forecasting by accomplishing two steps. The first step involves specifying the prior distribution for individual model parameters reflected by the error variances through the assumption of independent inverse Gamma priors. In the second step, posterior distributions are estimated using Markov Chain Monte Carlo simulation. To statistically ascertain the relative efficiency of the competing models, the present work deploys Diebold–Mariano (DM) test for equal predictability analysis. The DM test is capable of evaluating the differences among multiple forecasting models in terms of the accuracy manifested through mean-squared residual. As its operations are driven by paired comparisons, it is necessary to fix the order of the constituents in the pair to comprehend the findings. The competing models are marked with an index number inside parenthesis to represent the order. When the test statistic emerges to be positively significant, the model, indicated by the number 2 in the parenthesis, is judged to produce statistically better forecasts than the model marked number 1. The appearance of negative significant test statistics indicates the opposite scenario, i.e., the model marked number 1 in parenthesis is statistically more predictable than the second. Finally, if the test statistic turns out to be insignificant, it is assumed that there exists no significant difference between the models in terms of predictability. Tables 11, 12, 13 report the outcome of the statistical comparisons of dynamic forecasting on three different setups.

**Table 11:** Comparison of predictive performance on the original dataset

	ARIMA (1)	SARIMA (1)	BSTSF (1)	MLP (1)	EEMD-LSTM (1)	EEMD-PROPHET (1)
ARIMA (2)	–					
SARIMA (2)	0.235^#^	–				
BSTS (2)	0.221^#^	0.214^#^	–			
MLP (2)	4.6296***	4.6185***	4.5877***	–		
EEMD-LSTM (2)	6.8823***	6.8798***	6.8580***	5.9058***	–	
EEMD-PROPHET (2)	6.8754***	6.8631***	6.8544***	5.6162***	0.226^#^	–

***Significant at 1% level of significance

^#^Not significant

**Table 12:** Comparison of predictive performance during COVID-19 pandemic

	ARIMA (1)	SARIMA (1)	BSTSF (1)	MLP (1)	EEMD-LSTM (1)	EEMD-PROPHET (1)
ARIMA (2)	–					
SARIMA (2)	0.209^#^	–				
BSTS (2)	0.214^#^	0.228^#^	–			
MLP (2)	4.8231***	4.8370***	4.8042***	–		
EEMD-LSTM (2)	7.2198***	7.2261***	7.1875***	6.2818***	–	
EEMD-PROPHET (2)	7.2014***	7.2093***	7.1836***	6.2639***	0.232^#^	–

***Significant at 1% level of significance

^#^Not significant

**Table 13:** Comparison of predictive performance on the surrogate dataset

	ARIMA (1)	SARIMA (1)	BSTSF (1)	MLP (1)	EEMD-LSTM (1)	EEMD-PROPHET (1)
ARIMA (2)	–					
SARIMA (2)	0.228^#^	–				
BSTS (2)	0.206^#^	0.225^#^	–			
MLP (2)	4.7478***	4.7089***	4.7266***	–		
EEMD-LSTM (2)	7.8740***	7.8563***	7.8824***	7.8641***	–	
EEMD-PROPHET (2)	7.8596***	7.8477***	7.8615***	7.8342***	0.231^#^	–

***Significant at 1% level of significance

^#^Not significant

The outcome of the DM tests clearly rationalizes the superiority of both EEMD-LSTM and EEMD-Prophet over the competing ones in all three setups. There exists no significant difference in performance between EEMD-LSTM and EEMD-Prophet. The MLP model transpires to yield comparatively better predictions than the competing models. Thus, the propounded frameworks can be suitably used for tracking financial stress in normal, new-normal, and highly volatile situations. It should also be noted that the execution time of all competing models never crosses 380 s which is extremely praiseworthy as the same can be afforded for practical purposes.

### Model interpretation

**Fig. 17:**
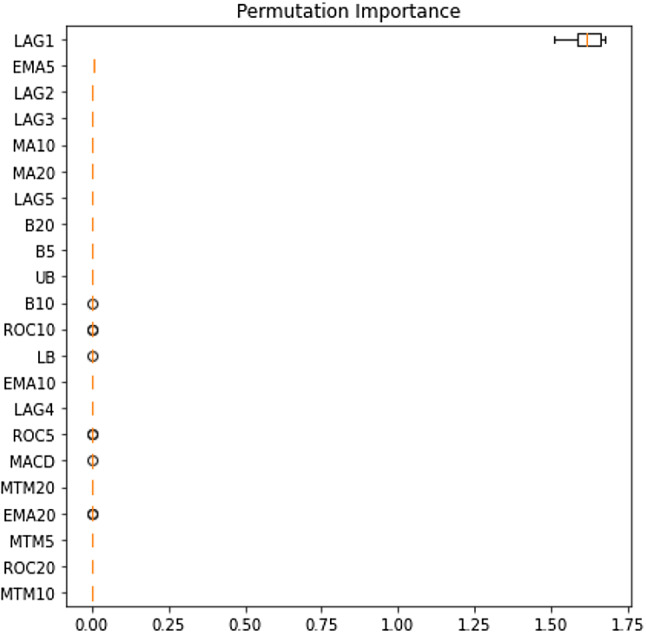
Permutation feature evaluation of the FSI prediction

**Fig. 18:**
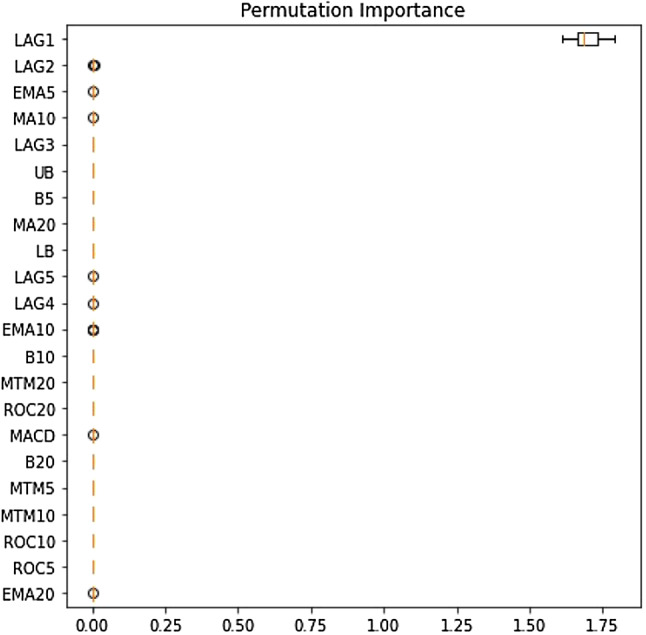
Permutation feature evaluation of the volatility prediction

To enable a deeper understanding of the nature of the impact of the chosen explanatory features, the XAI-based frameworks have been utilized. Initially, the outcome of the permutation feature evaluation is provided. Figures 17 and 18 display feature ranking for FSI and Volatility as samples.

**Fig. 19:**
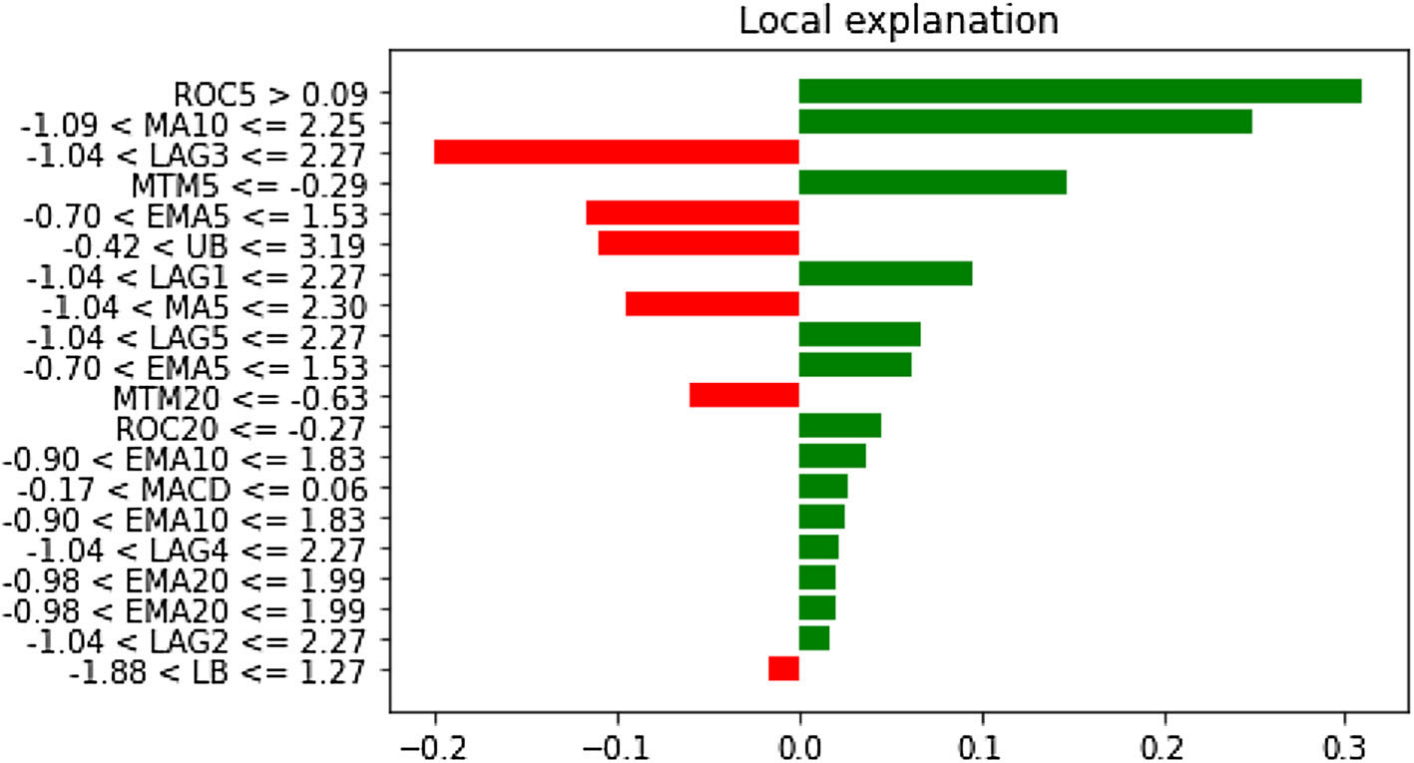
Local level feature contribution for the FSI prediction

**Fig. 20:**
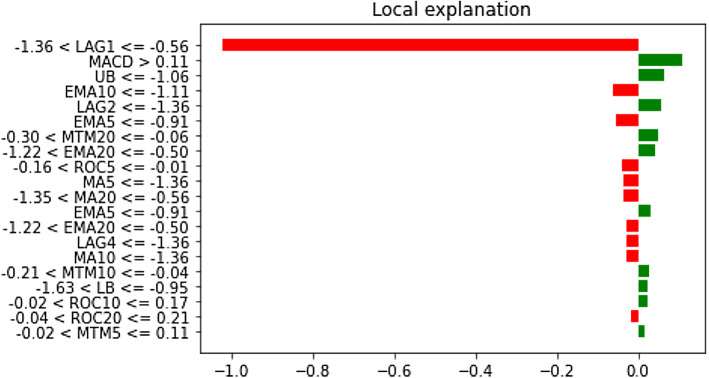
Local level feature contribution for the volatility prediction

It can be clearly observed that LAG1, LAG2, and LAG3 have occupied places in the top five important feature lists. The dominance of LAG1 in driving both stress indicators is also apparent. Therefore, it is evident that the immediate historical state of both variables largely explains the futuristic movements. The exercises have been extended to the remaining financial stress series too. Identical to these two variables, the prominence of LAG1, LAG2, and LAG3 in exerting significant predictive influence on those variables have been uncovered. Detailed results are available on request to authors. We then move forward to examine the interplay at the local level using the LIME framework on a randomly selected data instance. Figures 19 and 20 plot the contribution pattern of the top twenty significant features for FSI and Volatility prediction.

Unlike the global feature importance findings, it appears that different technical indicators barring the LAG1, LAG2, and LAG3, are critical to explaining the short-run dynamics. Several features, viz., ROC5, MACD, etc., which did not feature in the top five crucial feature list, have appeared to largely influence the local-level variation. Thus, in the context of static forecasting, it is of utmost importance to thoroughly track other features on top of the immediate past information. The magnitude of contribution is also dynamic. Similar results have been found for the remaining financial stress series, which are available on request to the authors.

## Conclusion

The paper attempts to develop integrated frameworks for inspecting the predictability of temporal dynamics of stress in global financial markets and key assets. As financial stress is a critical financial variable that shares a close association with other assets, recognizing the behavioral pattern to forecast future figures of the same becomes essential. The work critically examines financial stress across nine categories of OFR. The time horizons of compiled datasets include the COVID-19 pandemic timeline to inspect predictability during the new normal. The dearth of previous research was a concern for selecting exogenous constructs. To overcome the challenge, current work relied upon technical indicators, which are predominantly used as explanatory features in stock market prediction. The features have further been filtered using a dedicated feature evaluation algorithm, Boruta. The methodological forecasting framework resorts to the EEMD technique in decomposing the considered financial stress series. Subsequently, the deployment of LSTM and Facebook's Prophet has emerged to be enormously successful in yielding forecasts of supreme accuracy. A rigorous assessment of forecast quality suggests although being characterized by a high degree of non-stationary and heteroscedasticity traits, time series reflecting financial stress are indeed predictable. The supremacy of the proposed structures has prevailed over several well-known forecasting techniques. The additional insights through the deployment of the XAI methodologies serve a deeper understanding of the dependence pattern of the chosen variables on technical indicators. Traders and investors can be benefitted by tracking the short and long-run movements accordingly. Hence, the research can be classified as providing a profound contribution to financial stress literature and the methodological front, as the paucity of combining the state-of-the-art predictive structures and XAI is pretty apparent. The overall outcome of the research suggests that financial stress can be anticipated and explained at a granular level. Decoding the indicators in extremely volatile regimes and circumstances duly justifies the seamless integration of two predictive architectures, EEMD-LSTM and EEMD-Prophet, with the XAI methodological approach.

Thus, the outcome of predictive exercises justifies the efficacy of the proposed research architecture and marks the present work as a significant contribution to the existing research gap. FSI, the stress in the other five categories, Credit, EV, SA, Funding, and Volatility, and in regions, US, OAE, and EM have therefore been identified to follow a recognizable pattern. It has also been revealed that financial stress in EM and OAE were comparatively more predictable, while US and volatility-related financial stress were less predictable. A deeper granular inspection perhaps is required to analyze the said phenomenon, which is beyond the scope of current research. It, nevertheless, is apparent that financial stress can be predicted in normal and new-normal time periods. Precise estimation of one-day and multiple-day ahead future figures of chosen indicators imply the future state of fear, uncertainty, and volatility can be anticipated and effectively leveraged for risk mitigation. Portfolio realignment can be performed with utmost precision and efficiency. Past information on the variables should be monitored thoroughly, while short-run variations can largely be determined by tracking all technical indicators systematically. Methodologically, the combination of Prophet and EEMD is neoteric and has been pretty effective in drawing forecasts. To the best of our knowledge, the said combination has not been used for predictive analytics elsewhere. The granular framework of both LSTM and Prophet, in conjunction with EEMD, can be considered statistically efficient frameworks for modeling financial stress.

The scope of the current work is confined to forecasting selected indicators reflecting financial stress. It is also important to measure the predictability of financial stress realized in particular country-specific sectors. Over time, it would be possible to compare the extent of predictability during pre and post-COVID-19 pandemic. As mentioned, a deeper level investigation will be carried out in the future to identify the factors responsible for varying degrees of predictability of considered assets. Comparative performance assessment studies of LSTM and Prophet can be made with other emerging state-of-the-art deep learning methods in the future as well. The designed EEMD-LSTM and EEMD-Prophet frameworks can be extended to predict the temporal dynamics of a wide foray of financial assets via an appropriate selection of relevant explanatory features.

## Data Availability

The data that support the findings of this study are available upon request.
